# Should We Protect Animals from Hate Speech?^†^

**DOI:** 10.1093/ojls/gqab013

**Published:** 2021-05-31

**Authors:** Josh Milburn, Alasdair Cochrane

**Keywords:** hate speech, hate crime, free speech, animal rights, animal ethics, animal law

## Abstract

Laws against hate speech protect members of certain human groups. However, they do not offer protection to nonhuman animals. Using racist hate speech as our primary example, we explore the discrepancy between the legal response to hate speech targeting human groups and what might be called anti-animal or speciesist hate speech. We explore two sets of possible defences of this legal discrepancy drawn from the philosophical literature on hate speech—non-consequentialist and harm-based—and find both wanting. We thus conclude that, absent a compelling alternative argument, there is no in-principle reason to support the censure of racist hate speech but not the censure of speciesist hate speech.

## 1. Introduction

Imagine a group—let us call them the ‘White Defence League’ (WDL)—who distribute leaflets in a residential area. In their leaflets, the WDL decry the ‘propaganda’ spread by anti-racists and argue that white people are morally superior to those of other races. They further argue that white people should prioritise other white people—even if that means ignoring the exploitation, suffering and death of non-white people. If the WDL were operating in a liberal state with laws against ‘hate speech’, it is not hard to imagine that its members would face criminal prosecution for distributing the leaflets.^1^ In the UK, for example, members of the WDL might face prosecution for the distribution of ‘written material which is threatening, abusive or insulting’, that, regardless of the intent of the distributors, is likely to stir up ‘racial hatred’.^2^

But now imagine a different group, which we could call the ‘Human Defence League’ (HDL). The HDL also distribute leaflets in that same residential area. In their leaflets, they decry the ‘propaganda’ spread by animal-rights and vegan activists, and argue that humans are morally superior to other animals. They further argue that humans should prioritise other humans—even if that means ignoring the exploitation, suffering and death of non-human animals. While the claims and arguments of the HDL are very similar to the claims and arguments of the WDL—except, of course, that they are decrying animals, rather than non-white people—the HDL would not be subject to censure under hate-speech laws in the UK or any other liberal state.

There is thus a discrepancy between the legal response we could expect to the WDL’s and HDL’s respective leafleting campaigns. Our question is whether such a discrepancy is justified in criminal law. As such, we put to one side the related but separate questions concerning, first, whether hate-speech laws are justified at all (more on this shortly); second, the morality of engaging in hateful speech (ie engaging in hate speech may involve many wrongs whether or not it is the law’s business); and, third, the possibility of non-criminal legal means for challenging hate speech.^3^ In this article, we will explore several potential justifications for the discrepancy, ultimately concluding that none of them are successful. This leads to the conclusion that, in principle, there is no reason to believe that members of the WDL should face criminal sanction while members of the HDL should not. Unless a better justification for the discrepancy can be identified, either both (in principle) should face sanction or neither should.

Before exploring reasons for endorsing the discrepancy in earnest, we want to note two things. First, we acknowledge that, for some readers, even asking this question is distasteful, given historical and contemporary uses of animalising and dehumanising insults against non-white people. In response, we first note that the purpose of this article is absolutely not to equate the moral worth of non-white people with that of animals. We acknowledge that there are important differences between humans (of all races) and animals, and the nature and kind of injustices they face. We are interested only in the narrow question of what relevant differences there might be between the two *for the purposes of hate-speech law*, including differences in the impact that hate speech directed at these respective groups might have. We further note that the power of animalising language rests upon the fact that animals are themselves marginalised. If animals were afforded the respect that we (the authors) believe they are due, these insults would lose their rhetorical strength.^4^ We finally note that this comparison is intended to be a starting point to explore comparisons between hate speech targeting human minorities—racial, gender, sexual, religious and so on—and what we might call anti-animal or ‘speciesist’ hate speech. The specific comparison is not an end in itself.

The second consideration that we want to acknowledge is that there is already controversy about how, if at all, states can justify laws against hate speech, as well as what constitutes hate speech. To reiterate, the purpose of this article is not to take a stance on the question of justification. And yet, in exploring possible rationales for the discrepancy outlined, we will naturally examine a range of possible justifications for hate-speech laws. And though we acknowledge that there are difficult questions about the line between ‘merely’ hurtful speech and speech that could fairly be called *hate* speech, we wish to put that question aside. We are here focusing on the core question of whether one can distinguish paradigmatic forms of hate speech—racist, sexist, homophobic, ableist, etc—from what we might call anti-animal or ‘speciesist’ hate speech. Thus, we are not going to attempt to offer a definition or conception of hate speech, speciesist or otherwise, beyond noting that ‘hate speech’ is often thought to include material that is neither (in the colloquial sense) *hateful* nor *speech*.^5^ For now, we simply ask that readers allow that the WDL’s actions would typically be viewed as hate speech, while the HDL’s—though structurally identical—would not.

But while a precise definition of hate speech is not to be offered, it is reasonable to ask what kinds of anti-animal speech are motivating this enquiry. Some current practices that strike us as *potential* examples of speciesist hate speech include the following. First—and this is the kind of case that inspired the HDL vignette—pro-animal-agriculture organisations (including governmental organisations) publishing literature or films claiming that great harms inflicted on animals should be tolerated or ignored for the sake of seemingly less weighty human interests. Second, articles written by avowedly speciesist critics of animal protectionism claiming that animals’ interests do not matter *simply because they belong to animals*. Third, the public campaigning of conservationists built upon explicit declarations that the lives and suffering of members of certain non-native animal species matter less than the lives and suffering of members of native animal species. While each of these instances has a clear analogy in the case of hate speech targeting humans, each also brings with it its own particular conceptual and normative puzzles, which there is not space to explore fully here. Though these examples help motivate the enquiry, it might be the case that not all do (or should) constitute ‘hate speech’ proper.^6^

This article advances by examining a variety of different ways in which the legal discrepancy in responses to the WDL’s and HDL’s leafleting might be justified. We split these into two groups. The first section explores what we refer to as ‘non-consequentialist’ defences of the discrepancy—in that they justify the differential treatment in terms unrelated to the *impact* that the speech has (or is likely to have).^7^ The second addresses defences of the discrepancy grounded in claims about harm resulting from hateful speech. Our conclusion will be that none of the reasons to defend the discrepancy canvassed are satisfactory, leading to the conclusion that either both the WDL and the HDL should be open to censure or neither should.

## 2. Non-consequentialist Defences of the Discrepancy

### A. Veracity

One way to differentiate between the speech of the WDL and the HDL is via the veracity of their claims. Perhaps the claims in the WDL’s leaflets are untrue, while the claims in the HDL’s leaflets are true. Or, alternatively, perhaps the claims in the WDL’s leaflet are not only false but indefensible, while the claims in the HDL’s leaflet—whether true or false—are at least defensible.

There are at least two problems with this line of reasoning. The first is that it is question-begging. The WDL and their supporters, at least, are presumably going to deny that their claims are wrong—and are certainly going to deny that their claims are indefensible, even if anti-racists may say that they are. Meanwhile, plenty of animal liberationists and anti-speciesists will deny that the HDL’s claims are right, and may be prepared to claim that they are indefensible. In response, it might be observed that decisions *must* be made about rightness and wrongness (or defensibility and indefensibility) for the idea of hate-speech legislation to have plausibility at all. That is, the untruthfulness (or indefensibility) of the WDL’s claims (as determined in some democratically appropriate way) might seem to be a prerequisite for labelling their activities hate speech.^8^ This response can be countered by observing that those whose status is disputed could be *more* in need of the protection of hate-speech legislation than are those whose status is assured (for more on this, see our discussion of Waldron below). It seems confused, for example, to offer the protection of hate-speech legislation to members of a dominant racial group on the grounds that all in the *demos* recognise the equal status of members of that group, but deny it to racial minorities on the grounds that many in the *demos* are racists. In effect, this gets the matter the wrong way around.

The second problem is that there is something deeply illiberal about censuring the expression of a view simply because the view is wrong, or even indefensible—or, more precisely, *deemed* to be wrong or indefensible. John Stuart Mill, for example, famously argues in defence of freedom of speech *regardless* of the rightness or wrongness of the expressed view.^9^ Censured views, he argues, may be true, meaning they should be adopted; may contain a portion of the truth, meaning they should be heard, so that prevailing opinion can develop; or may be untrue, meaning they should be heard, so that the truth of the received opinion may be earnestly felt, and the received opinion does not become mere ‘dead dogma’. And, in any case, we have no way of *knowing* whether views are untrue until they have been heard. (And we cannot say that we know they are wrong because we have heard them before; we have no way of knowing that we have heard them before without hearing them this time.) For Mill, the state can interfere with an individual’s freedom of speech—indeed, any action of an individual—only when the interference will protect others from (wrongful) harm.

With a few notable exceptions, Mill does not believe that the expression of an opinion can be harmful—and he is unlikely to see the actions of the WDL or the HDL as harmful.^10^ (We may want to challenge this view, and we will explore reasons for thinking that the WDL’s actions are harmful later in the article.) But let us put aside the suggestion that the discrepancy between the WDL and the HDL can be justified by the accuracy or defensibility of their respective views.

There is, perhaps, a more sophisticated version of this argument that says that speciesist speech (even if untrue) is within the space of reasonable or legitimate public debate, while racist speech is not. This idea is usefully explored through the idea of public reason. John Rawls holds that the burdens of judgment (the challenges leading reasonable people to make different judgments about ethical matters) and the consequent fact of reasonable pluralism (the existence of a plurality of reasonable views) mean reasonable disagreement will persist in a liberal society.^11^ So, Rawls (himself a vegetarian) might have said that reasonable people will continue to disagree about the moral worth of animals. But there are some things that no reasonable person would disagree with, such as the equal moral status of human beings; these are not the subject of reasonable disagreement in a liberal society. What is more, human rights are a matter of basic justice, while the rights of animals are not. This gives us a clear difference between the actions of the WDL and the actions of the HDL. The HDL are contributing to legitimate public debate about a controversial issue, one that steps beyond matters of basic justice into (understood in a Rawlsian sense) metaphysics. The WDL, on the other hand, are not making such a contribution: the things they are discussing are beyond reasonable disagreement, and are a matter of basic justice.

Drawing on this distinction, one could construct argument saying that while the state has no business restricting freedom of speech—a Rawlsian basic liberty—when the speech concerns a matter of reasonable disagreement, speech on matters beyond reasonable disagreement could, in principle, be restricted.^12^ Thus, the state could legitimately restrict the WDL’s speech but not the HDL’s speech.

We believe that there are two important responses to this position. The first would be to simply regard Rawls’s specific conceptions of public reason and basic justice as inappropriate for the present question. After all, the idea that animals’ rights are a subject of public debate while humans’ rights are not is built into the idea of public reason and ‘reasonableness’. Federico Zuolo explains that the idea of public reason presupposes that human persons (to whom positions must be justified) have rights, but leaves as an open question whether animals have rights.^13^ But such presuppositions seem to simply stack the deck against animals. The alternative view is that we need to ‘accept a *new* form of reasonableness’, according to which views not sufficiently respectful of animals are deemed *un*reasonable, just as views not sufficiently respectful of members of racial minorities are.^14^ This need not mean that Rawls was *wrong*, but simply that standards of reasonableness can change.^15^ Maybe we are now more aware of the status of animals, or perhaps instead what the animal advocate must hope is ‘for a sufficient consensus on the worth of animals to emerge at some point in the future’.^16^ If that is the case, then the present article can be read as philosophy of law for a more ideal world. (This thought will be returned to below.)

An alternative response would be to accept (something like) Rawls’s conception of reasonableness, but maintain that views regarding animals as lacking *any* status are unreasonable.^17^ And, if the HDL are spreading *these* views, then their conduct is (according to the present line of thought) somewhat analogous to the actions of the WDL. It has been argued, for instance, that opposition to animal cruelty is a kind of fixed point in public reason^18^ (much as anti-slavery is a fixed point) or that animal welfare laws are supported by an overlapping consensus^19^ (much as human rights are supported by an overlapping consensus). Rejecting *these* things could thus be a kind of anti-animal hate speech. Consequently, on this alternative response to the public-reason argument, while *some* examples of speech that could be labelled ‘hateful’ towards animals would be justifiable and within the realm of public reason, others would not. *Some* instances of supposed anti-animal hate speech would therefore warrant criminal censure analogous to the criminal censure warranted by racist hate speech (if any), but other instances of supposed anti-animal hate speech would not.

For present purposes, we are not interested in adjudicating between these two responses. Our point is simply that while the public-reason case *may* point to disanalogies between the actions of the HDL and the WDL, it does not justify rejection of the idea that animals warrant the protection of hate-speech laws (if humans do).

### B. Race and Species Are Not Analogous

Another means by which some might defend the discrepancy would be to argue that while the *form* of the speech of the WDL and the HDL is comparable, the *content* of their speech is completely different. Non-white people (like members of some other denigrated groups in society) are appropriately protected by hate-speech laws because of the kind of thing race *is*; members of denigrated species are not protected, on the other hand, because of the kind of thing species is. For this reason, race and species are not analogous. (Note that this is a different argument to that discussed in the previous subsection. It concerns not the rightness or wrongness of the positions of the WDL and the HDL, but the different kinds of things they are making claims about.)

We acknowledge that race and species are not the same thing—but note that the existence of difference does not undermine the possibility of making certain legitimate analogies between them. And let us remember that hate-speech laws *already* protect people from hateful speech targeting many different characteristics. Depending on the jurisdiction, for instance, they may protect individuals from abuse directed at their religion, sex, sexual orientation, physical or mental abilities, and so on. The pertinent question is whether there is a *morally salient* difference between race and species that justifies treating race, but *not* species, as a protected characteristic under hate-speech law. If there is, it will be due to a ‘formal’ specification of the kinds of groups that are appropriately protected by hate-speech laws.

Drawing upon existing scholarly and legislative discussions, Alexander Brown identifies five formal specifications used to delineate the characteristics worthy or unworthy of protection in hate-speech law: (i) immutable versus changeable characteristics; (ii) chosen versus unchosen characteristics; (iii) constitutive versus peripheral characteristics; (iv) internal-life versus external-life characteristics; and (v) characteristics we all share versus characteristics we do not all share.^20^ The idea is that these distinctions provide (in the eyes of their advocates) morally salient ways to delineate traits that are eligible for protection via hate speech laws with reference to features *of the traits themselves*. For example, in the eyes of some commentators on hate-speech legislation, it would be non-arbitrary to protect people from hate speech targeting them as members of a group with a particular immutable characteristic (eg people born in a particular country), while not protecting them from (putative) hate speech targeting them as members of a group with a particular mutable characteristic (eg people employed by a particular company). If it were the case that race and species fell on different sides of these various distinctions, then we might have an in-principle justification for the discrepancy in responses to the WDL and the HDL. As such, these formal criteria are worth reviewing.

Two distinctions can be put aside relatively easily. The idea that a characteristic that is (relatively or absolutely) changeable (eg religion) is less eligible for protection than a characteristic that is (relatively or absolutely) immutable (eg sex) will not serve to distinguish species from race. It seems clear that just as one cannot generally go through multiple races in one’s life, one cannot generally go through multiple species; both are, then, ‘immutable’. The distinction between ‘chosen’ versus ‘unchosen’ characteristics also fails to distinguish species from race, for while some might claim that (say) political affiliation is less worthy of protection in hate-speech laws than (say) race, on the basis that the former is (largely) chosen and the latter is (largely) unchosen, the distinction does not apply to *species* and race. Just as we do not generally choose our race, so we do not and cannot choose our species.

The internal life versus external life distinction pushes in the opposite direction to those addressed in the previous paragraph. One’s ‘inner life’ concerns ‘inner thoughts, feelings, beliefs, desires, and even understandings of the meaning of life and of the type of people it is good to be’.^21^ Thus, one’s religious belief (minimally, an element thereof) is a paradigm example of an inner-life characteristic. One’s external life, on the other hand, ‘has to do with outward appearances, and with how people are presented to, and interact with, other people’.^22^ Thus, one’s race is a paradigm example of an external-life characteristic. Somewhat in contrast to the conclusions reached above, advocates of this distinction present external-life characteristics as *less* worthy of the legal protection afforded by hate-speech legislation than internal-life characteristics. Nonetheless, it seems clear that this distinction is not useful for distinguishing species from race: both are paradigmatic external-life characteristics.

Another distinction is sometimes made between characteristics we all share and those that we do not. This concerns the difference between those classificatory schemata for which most of us will pass through the various classifications and those schemata for which we will not. For example, most of us, at different times in our life, will be children, young adults, middle aged, and elderly. Most of us will not, at different times in our life, be Christian, Jewish, Muslim, Sikh, Hindu, and Buddhist. Again, though, this specification is no good for defending the discrepancy: both race and species fall on the ‘unshared’ side. All of us have both a species and a race, but these stick with us throughout our lives—or, if they do not, it is generally because things change around us. (Both species and race classifications are fluid in the sense that members of a species or a race are subject to being reclassified by others.) And while people can and do identify (or be identified) with multiple races, similar can be true of species. For example, scientists disagree about species classifications, some individuals seem to fall between multiple species (such as interspecies hybrids, members of ‘ring species’, etc), and so on. There is no one who will be or is all or most races—and there is no one who will be or is all or most species.

The most plausible basis for distinguishing race and species as characteristics for the purposes of hate-speech legislation, then, is on the grounds that one of them is a ‘constitutive characteristic’ and one is a ‘peripheral characteristic’. Specifically, we might argue that race is constitutive of an individual’s identity, while species is not. The argument may go that species membership is like whether someone’s ears are attached, or whether she can roll her tongue. These are features that we have, and features that may distinguish us, but (unlike race) they are not features that make us *who we are*. The first thing to say is that the argument cannot be that race is constitutive for *all* individuals. Plausibly, there are some individuals for whom race is *not* a significant part of their identity, and that fact alone cannot be sufficient to disqualify hate-speech laws censuring racist speech. Nor can the claim be that *only* those for whom race is a constitutive part of their identity warrant protection from racist hate speech. It is unclear how this would work in practice, and it has some deeply undesirable consequences. Iris Young, for example, uses the example of Jews in Vichy France who had lost their identity as Jews, but who were starkly reminded of it when faced with Nazis.^23^ The concept of *thrownness*, which Young borrows from Martin Heidegger, is instructive. We find ourselves *thrown into* identities and positions. It is perverse to suggest that these Jews would not warrant the protection of hate-speech laws. Similarly, it would be perverse to suggest that very young children without any sense of their race are not entitled to the protection of hate-speech laws. Consequently, the argument must be that race is a constitutive part of the identities of some significant number of individuals, while species is not. It could be this that makes it appropriate to censure the WDL, but not the HDL.

But, even if we accept its salience for the sake of argument, constitutiveness fails to distinguish species from race: quite simply, species identity *is* constitutive to the identities of many individuals. Many animals clearly recognise themselves as belonging to a particular kind—birds of a feather, as the saying goes, flock together. This is obvious, and perhaps best demonstrated by its exceptions. Stories of dogs who ‘think’ they are cats are charming, while stories of narwhals swimming with belugas are intriguing. The norm, of course, is that dogs ‘think’ they are dogs, cats ‘think’ they are cats, narwhals swim with narwhals, and belugas swim with belugas.

The defender of the discrepancy might say that this does not prove anything about the *identity* of these animals—no dogs *identify* as dogs or cats, no narwhals *identify* as narwhals or belugas. But there is clearly at least one kind of being for whom species membership is frequently constitutive of identity: humans. Anthropocentric attitudes are at the core of many religious understandings of what makes us *us*, and, indeed, many *non-*religious conceptions of the same. It is, perhaps, no coincidence that many people reject religious conceptions of the world by affirming *human*ism.

Let us summarise this subsection. It might seem obvious that race and species are not analogous for the purposes of hate-speech legislation, and so that a ‘formal’ difference between them can justify a difference in treatment in hate-speech law. We have canvassed five ‘formal’ reasons to exclude certain characteristics from the protection of hate-speech law. Four evidently do not serve to distinguish species and race as characteristics. A fifth might: it may seem that race is constitutive of the identities of many, while species is not. This, however, does not stand up to scrutiny: species, too, is core to the identities of many individuals. Unless other formal criteria can be identified to catergorise race and species as characteristics (respectively) appropriately warranting and appropriately *not* warranting the protection of hate-speech law, this defence of the discrepancy fails.

What this subsection has *not* demonstrated is that race and species are analogous for any purpose other than this. Nor has it demonstrated that there might not be other—non-formal—reasons to differentiate race and species for the purpose of hate-speech legislation.

### C. The Targeted Groups Are Not Analogous

It could be that there is a disanalogy between the WDL and the HDL, but that this comes down to the differences between the *particular* race/species groups targeted, and not differences between the categories of ‘race’ and ‘species’. So, to use an unrelated example, it could be that women are appropriately protected by hate-speech legislation while men are not. Although, *in formal terms*, the groups ‘men’ and ‘women’ are analogous, the way that members of the groups are (or historically have been) treated may constitute a relevant difference between them. Our question is the following: are there important differences between the group ‘non-white people’ (as targeted by the WDL) and the group ‘non-human animals’ (as targeted by the HDL) that justify protecting the former, but not the latter, using hate-speech legislation?

Again, we acknowledge multitudinous differences between these two groups—but (again) the existence of difference does not undermine the possibility of drawing analogies. The pertinent question is whether there is a *salient* difference between the groups that justifies treating one, but not the other, as warranting the protection of hate-speech laws.

When seeking objective means for selecting groups that warrant protection under hate-speech law beyond formal criteria, we can borrow from the broader literature on hate crimes, which adopts two strategies.^24^ The first is to identify *minority* groups; the second is to identify *vulnerable* groups. The question, then, is whether the group targeted by the WDL could fairly be differentiated from the group targeted by the HDL on the grounds of minority status or vulnerability.

In this literature, and more broadly in the social sciences, *minority* does not refer to relative numbers; instead, the focus is on the structural inequalities faced by the group. So, while national or ethnic minorities are paradigm examples, because of the structural inequalities experienced by members of different groups, talk of women (*qua* women), LGBTQ people (*qua* LGBTQ people), or disabled people (*qua* disabled people) as members of minorities is now relatively familiar. But if we define ‘minority’ relative to structural inequality, it is hard to see why animals should not be included.^25^ Animals certainly seem to be captured, for example, by straightforward definitions of minority groups as ‘social groups that are oppressed or stigmatized on the basis of racial, ethnic, biological, or other characteristics’.^26^

But, it may be objected, animals are not captured by more sophisticated definitions of minority groups. A classic account comes from Louis Wirth:

A minority group is any group of people who, because of their physical or cultural characteristics, are singled out from the others in the society in which they live for differential and unequal treatment, and who therefore regard themselves as objects of collective discrimination.^27^

The problem is twofold. First, many might claim that animals are excluded from this understanding because they are not people, since *people* means ‘humans’. But such a move seems overly stipulative and *ad hoc*. Why should the definition be limited to humans in this way? For the purposes of this definition, at least, it seems just as reasonable to suppose that animals are people, too. Second, animals may seem to be excluded because they do not, so far as we know, ‘regard themselves as objects of collective discrimination’. But this is a problematic way to limit the definition, for two reasons. The first recalls Young’s words of the previous subsection: individuals can find themselves *thrown* into a group, discovering that they have always already been a member, even if they did not realise it. This means that one can *be* a member of a group without *regarding oneself* as a member of the group (or as the object of collective discrimination). Second, just as animals cannot conceive of themselves as objects of collective discrimination, so many humans cannot conceive of themselves as such. For example, very young children lack awareness of discrimination they face, but we nonetheless would not want to deny that they can be members of a minority group. Thus, the fact that animals fail to meet the letter of Wirth’s account of a minority group should not stop us identifying them as such.

We conclude that the notion of a ‘minority group’ is not a helpful one for justifying the discrepancy between the treatment of the WDL and the HDL, and turn to our other candidate criterion.

Could it be, then, that non-white people are members of a *vulnerable* group, while animals are not? Joanna Botha, drawing upon Jon Garland,^28^ identifies vulnerable groups by asking

whether the group (a) is a distinct social outgroup, (b) which endures repeated targeting as victims of abuse or discrimination, (c) is prone to stranger crimes, and (d) experiences the impact of hate crimes in a way which undermines the social confidence of the individual victims.^29^

Nonhuman animals clearly represent a distinct social outgroup (a), and clearly face repeated violent abuse and discrimination at human hands because of their membership in that outgroup (b). Animals are prone to stranger crimes (c) insofar as they often face illegal violence at the hands of humans they have never met before—for example, cows abused by overworked slaughterpersons, wild animals poached, or companion animals poisoned by those who regard them as pests.

But do animals experience the impact of hate crimes in a way that undermines their ‘social confidence’ (d)? At first sight, it might seem not. This could justify the discrepancy between our reactions to the WDL and to the HDL: the former target a vulnerable victim group, the latter do not.

There are two responses that can be offered. The first is that *some* animals do seem to have their social confidence eroded because of their awareness of the risk of violence. ‘Game’ animals who learn to avoid humans or companion animals who have suffered abuse could be described as individuals whose social confidence has been eroded because of fear of attack. If correct, this would mean that (at least some) animals are part of a vulnerable group, warranting relevant protective laws as such. The second response echoes concerns stated previously. There are *some* humans who lack the cognitive capacities for their social confidence to be eroded by knowledge of hate crimes. Mental illness, developmental disability, or neuroatypicality, for example, could result in individuals being impacted by knowledge of hate crimes (if any) in ways different to many other adults. But it would be a surprising, even disturbing, conclusion that these individuals cannot make up vulnerable victim groups for the purposes of hate crimes. After all, it is well established that the mentally ill, the disabled, and neurodivergent individuals are the victims of hate crimes. The fact that some mentally ill, mentally disabled, or neurodivergent individuals may experience knowledge of hate crimes (if any) differently to others should not change that. Similarly, the fact that animals may not be impacted by knowledge of their membership in a vulnerable group should not stop us recognising that they are vulnerable.

This subsection has asked whether the fact that non-white people are members of a ‘minority group’ or a ‘vulnerable group’, while animals are not, could justify the discrepancy. We have concluded that it cannot.

## 3. Harm-Based Defences of the Discrepancy

### A. The Harm of Wounding

Perhaps the most obvious type of harm putatively inflicted by hate speech derives from the psychological distress and mental pain it can cause. As Richard Delgado famously put it, there are some ‘words that wound’.^30^ For example, racial slurs, epithets, and insults can lead to humiliation, isolation, and self-loathing. Furthermore, these psychological effects often result in further harms through their impact on bodily health and livelihoods. If this kind of ‘psychological wounding’ is an important harm of hate speech, it could be a means to differentiate the WDL and the HDL: the actions of the former cause mental anguish to non-white people, but the actions of the latter cause no such wounding to animals.

The feature of the wounding words approach that offers support for the discrepancy is its subjectivity. Taking the wounding-words account as given, we see that, from the perspective of (actually existing) animals, the HDL’s actions are non-wounding, as they are not comprehensible.^31^ From the perspective of (many) non-white people, however, the WDL’s actions are both comprehensible and wounding. But while the account’s subjectivity can support the discrepancy, it can also introduce problems.

First, there are what we might call ‘false negatives’—victims of (what seems to be) hate speech who are nonetheless not wounded. We say this because (both in fact and in theory) some targets of hate speech are not wounded by the words, but are instead unaware of them (or aware of them but merely angered, motivated, or amused). It would be bizarre and impractical to say that wounding words are appropriately censured only if they *actually* wound—among other problems, this would mean that even the most egregious instances of hate speech would be permissible if, for whatever reason, no one was hurt by what was said.

The significance of this is that the WDL could, in theory, conduct a leafleting campaign in such a way that no one who would be wounded by their leaflets was given one and those who received the leaflets kept this fact to themselves. Nonetheless, intuitively, their leafleting remains problematic. The advocate of the wounding words approach could bite this bullet, allowing that this WDL leafleting campaign should be permitted. We suspect, however, that they would not want to. And refusing to bite this bullet could have consequences for anti-animal hate speech.

One way that a wounding approach could deal with the challenge of false negatives is to introduce something like a test of ‘hypothetical universalizability’.^32^ This would mean noting (to adapt Joel Feinberg’s words, first used in a different but related context) that people would be wounded by leaflets targeting ‘*their own* race, religion, or ethnic group’,^33^ and thus allowing (again paraphrasing Feinberg) that

When a contemplated action is predictably likely to wound virtually any person who might happen to behold it (or would wound nearly any person *who found himself the target of a similar affront*, when the wounding is aimed more narrowly), then there is a very powerful case for forbidding it.^34^

While this satisfactorily overcomes the problems of false negatives, it also opens the door to protecting animals from the HDL’s leafleting. Given hypothetical universalisability, any anti-animal hate speech that would be wounding to the audience *if animals were replaced by some minority group of which they were members* would be as serious as the speech that *actually* targets the minority group of which they are members. Thus, if hypothetical universalisability is accepted, the wounding approach is powerless to differentiate between the WDL and the HDL, *even if* (as it happens) animals are not wounded by the HDL’s speech.

Might there be other ways for the wounding-words account to deal with false negatives? One possibility would be to hold simply that words deemed *sufficiently likely to wound* should be subject to censure. This strategy could avoid biting the bullet that a non-wounding HDL leafleting campaign is legitimate. At the same time, and unlike the invocation of hypothetical universalisability, it would mean that animals would *not* be protected. After all, we can be sure that animals will not be wounded by words that they cannot understand.

But the success of this strategy in dealing with false negatives is mixed. It can deal with *some* false negatives, such as those cases in which no one is wounded by some fluke. But there are other false negatives that this ‘sufficiently likely to wound’ approach cannot resolve. So, for example, if the WDL deliberately planned a leafleting campaign so that they could spread their literature *only* to white people sympathetic to their message, they could argue that not only was no one *actually* wounded, no one was *likely* to be wounded. And, to draw upon a recurring concern, many young children and people with various disabilities will not understand (and thus not be wounded by^35^) hateful speech.^36^ Thus, an advocate of the wounding words approach could justify the discrepancy, but at considerable cost. This leads us to believe that the more defensible version of the wounding words approach would draw upon hypothetical universalisability—and thus, in principle, protect animals.

There is another side to the puzzle of the wounding-words account’s subjectivity. This is the question of ‘false positives’—cases in which individuals (quite predictably) experience psychological ill effects from speech acts that could not reasonably be described as ‘hateful’ (in the technical or colloquial sense). Take, for example, rejections in the romantic arena.^37^ We do not raise this further concern with the account’s subjectivity because we are arguing that the wounding-words approach should be rejected. Such a claim is obviously beyond the scope of this article. We simply note that, in responding to this puzzle, we are pushed towards making an identification of wrongful action not on the subjective feelings they produce, but on the *wrongful content* of the speech itself.^38^ (The importance of this, of course, is that it is a step away from the very subjectivity that allowed the wounding-words approach to offer a foundation for the discrepancy in the first place.) But any more objective approach seems to immediately run into difficulties. We have already seen that to claim that the HDL’s speech is more defensible or truthful than the WDL’s is to beg the question. The next subsection thus reviews a different way in which to identify an objective basis for the harms caused by the WDL and HDL.

### B. The Harm of Inculcating a Hostile Environment

Some scholars have argued that the real harm in hate speech is indirect.^39^ Hateful speech acts can create a ‘hostile environment’, which acts as a ‘tinderbox’ not only for violent attacks and assaults, but other important harms, too. Furthermore, if these speech acts can take place without challenge, they may be normalised, thereby encouraging others to engage in similar behaviours.^40^ The resulting climate of hatred can

cause members of the targeted group to be more likely to face discrimination in the work place, to be excluded from the political process and the top positions in society, to be ignored in matters of social justice, to find it more difficult to gain access to education, housing and health care, to be more likely to experience mistreatment at the hands of the police, to suffer miscarriages of justices, and so on.^41^

Perhaps we can differentiate the WDL from the HDL, then, by saying that the WDL’s speech creates and sustains an environment that is conducive to violence, discrimination and other forms of injustice, whereas the HDL’s does not.

Of course, the problem with identifying the harm of hate speech in this way is that it is very hard to substantiate empirically. For one, it is extremely difficult to know that any speech acts do cause the creation and maintenance of this kind of climate of fear, rather than other factors.^42^ Furthermore, for our purposes, we have no way of knowing that the WDL’s speech will create a more hostile environment for its targets than the HDL’s, for while members of racial minorities suffer seriously from violence, discrimination and injustice, so too do animals. Indeed, *tens of billions* of animals are bred, confined in terrible conditions and slaughtered in infancy every year throughout the world.^43^ This is exactly what the leaflets circulated by the HDL—and real-world groups like them—seek to defend. Given this, there seems no obvious way by which we can say that the causal links between speech acts and a hostile climate are clearer for racist speech in comparison to speciesist speech. Perhaps, if anything, anti-animal speech causes even *more* suffering overall. For example, the HDL’s leaflets encourage people to be unworried about everyday practices that already routinely contribute to considerable harm to animals (for example, through buying products produced via intensive animal agriculture). The WDL’s leaflets, on the other hand, encourage attitudes and practices which are not widely shared in most communities, and hence are far more likely to ignored.

One could object that there is something bizarre about challenging the creation of a hostile environment for animals when animals are already treated so badly at human hands—when we are already, in Dinesh Wadiwel’s words, waging a war against animals.^44^ Animals, perhaps, have little to lose by the creation of an environment hostile to them, while members of racial minorities have a great deal to lose by the creation of an environment hostile to them. We offer a two-part response to this objection. First, while animals are routinely exploited in all contemporary societies on a massive scale, things *could* get worse for them. For example, additional animal farming operations could be rolled out, processing even greater numbers of animals. Furthermore, the intensity of the practices within those operations could increase, perhaps through politicians rolling back existing (modest) animal-welfare protections. Second, we accept that this article might usefully be read as an argument suited to a more ideal world: that is, one in which our war against animals has come to an end. If so, then we are perhaps asking what *future* laws governing human/animal relationships might look like, as was noted earlier. And yet, as will be explored later, instituting bans on speciesist hate speech might help inculcate more benign human–animal relations within society; to put it another way, such bans may be part of our *route* to that more ideal world.^45^

### C. The Harm of Denying Membership

Jeremy Waldron’s theory of hate speech draws on the ideas of a wounding and hostile environment, but develops them in interesting ways. Crucially, his claims about the ‘harms of hate speech’ provide two important possible resources for differentiating the WDL from the HDL. First, and in keeping with the hostile environment claims discussed in the previous subsection, Waldron explicitly attempts to ground the harm in hate speech in something *objective*. For him, the harm lies in the fact that such speech denies an individual’s equal status or civic ‘dignity’.^46^ Hate speech, for Waldron, comprises those forms of communication that express intense antipathy towards some group, or towards individuals because they share some affiliation with that group.^47^ It is harmful, according to Waldron, because it undermines the recognition of those individuals as equal members of the community. He thus sees hate speech as a form of ‘group defamation’: it not only denigrates certain individuals on the basis of their belonging to certain groups, but attacks their reputation as individuals with equal membership, rights, standing, and belonging in that community.^48^

But why does Waldron regard the harm caused by this assault on ‘dignity’ to be a proper basis for state sanction? This comes down to Waldon’s vision of what a ‘good society’ requires. He argues that a ‘well-ordered society’ must formally acknowledge the equal standing of all its members. However, he also claims that it must go further than this; members not only have an interest in possessing formal status and rights, but also in being *treated* as if they have that status and those rights.^49^ The ‘assurance’ that we get from knowing that we can go about our business without being humiliated and denigrated is an important public good, according to Waldron. Crucially, he points out that it is especially important for members of those minority groups who have been openly despised by some in their community in the past, and who have only attained their formal equal standing recently.^50^ For this reason, Waldron argues that we all have a duty to establish and maintain a ‘visible environment’ that explicitly and implicitly provides this assurance. As such, the state has a duty to prevent those forms of speech that assault the dignity of some of its members.^51^

At first blush, it might appear as though Waldron’s theory provides a plausible basis for differentiating between the WDL and the HDL. For one, animals are not individuals with equal social standing in any community, thus they do not possess the political status that Waldron thinks hate-speech laws ought to protect.

In our view, however, this is too hasty: the best reading of Waldron’s theory must include certain animals within its protective remit. To explain, let us acknowledge the fact that no political community recognises animals as members with equal social standing. In all existing political communities, animals have a second-class status in comparison to humans. That can mean that animals are mere property—commodities that can be bred, raised, confined, traded, and killed for the benefit of humans.^52^ And even among those jurisdictions that have explicitly declared that animals are more than mere property,^53^ that have declared that they have a value of their own as sentient creatures^54^ and have enacted animal-welfare laws restricting certain harmful ways in which animals can be treated, none has come close to prohibiting the horrors of intensive animal agriculture, let alone recognising that animals have equal standing in the community. It is further true that some states have enacted constitutional provisions for the sake of animals, some of which explicitly recognise the ‘dignity’ of animals.^55^ But, again, none of these provisions acknowledges that animals possess the Waldronian sense of civic dignity: none views animals as possessing equal social standing, membership, status, and rights. No community truly regards its animal residents as *members* of society, and none recognises them as *equals*.

It certainly seems, then, that the HDL’s leafleting does not harm animals in the way Waldron is interested in; it does not assault their dignity, for the simple reason that animals do not possess dignity as Waldron defines the term. But while we believe that this is the conclusion that Waldron himself would likely draw in relation to hate speech and animals,^56^ we do not believe that it is correct. One question that is immediately raised by this analysis is whether animals *should* have the kind of dignity that Waldron puts at the heart of his theory of hate speech. While they might not be regarded as members of equal social standing, should they be? One influential argument claiming that they should has been provided by Sue Donaldson and Will Kymlicka.^57^ For Donaldson and Kymlicka, domesticated animals should be regarded as equal members of our communities. Unlike wild animals, many of whom live apart from us in their own communities, domesticated animals have been deliberately brought into our communities to live, work, play, rest, and die alongside us. Animals are a central feature of our ‘multi-species communities’, providing care, security, companionship, food, and more to ensure the flourishing of the societies in which they reside. Furthermore, these domesticated animals have been bred over the centuries to possess certain characteristics that are beneficial for humans. This means that these animals are usually dependent upon humans for their own well-being. But, unlike those ‘liminal’ animals who live within and amongst human dwellings—birds, rodents, foxes, and more—it also means that domesticated animals can be socialised and adjust their behaviour to conform to certain social norms and rules. As a result of these facts, Donaldson and Kymlicka claim that domesticated animals are entitled to more than simply being recognised as ‘sentient beings’ with a moral status that we have a negative duty not to impinge upon. Rather, they argue that domesticated animals should be recognised as the members of society that they are, with the relevant status and rights that entails.

Crucially, one need not accept every element of Donaldson and Kymlicka’s theory to include certain animals within a Waldronian theory of hate speech. For one, we might accept that domesticated animals ought to have equal membership, even while denying them certain civil rights. This is because, while Donaldson and Kymlicka believe that animals’ membership should include recognition of the rights of ‘citizenship’, such as rights to be involved in the co-authoring of public policy, we can reject this claim while embracing animals’ equal standing. Put simply, we can acknowledge that different individuals in a society are equal in their possession of status and rights, even when the rights they hold might be quite different. The case of infants is instructive here: awarding children a set of rights different to those possessed by most adults^58^—say, by denying them rights to vote or to marry and granting them rights to schooling and adequate parenting—does not entail rejecting their equal membership. Alternatively, we might accept that some animals ought to enjoy features of social membership while nevertheless denying them *equal* social standing. Perhaps, and contrary to Donaldson and Kymlicka, a hierarchical scheme of standing is the more appropriate way of recognising animal membership. But even if this is true, a Waldronian theory of hate speech should still protect those animal members—even if in a less stringent way than the protection offered to full human members. After all, a denial of animals’ membership would still constitute an assault and a harm that it is the law’s business to address.

Furthermore, whether one follows Donaldson and Kymlicka in attributing equal membership to animals or develops a hierarchical conception of membership, membership might be limited to only *certain* animals. For while Donaldson and Kymlicka grant membership to all domesticated animals, and some go even further to include all sentient animals,^59^ it is possible to take a more parsimonious view. For instance, some argue that only those domesticated animals most clearly and closely involved in our schemes of social cooperation can meaningfully enjoy membership.^60^ Indeed, this more minimalist understanding of animals as social members has some legal precedent in several states.^61^ So perhaps only a relatively small number of animals—for example, companion animals and those working alongside humans in cooperative projects—ought to benefit from protection under the laws of hate speech.

What matters for our purposes is not resolving these debates about the bundles of rights entailed, equal or hierarchical membership, or the animals included. Rather, what matters is the mere fact that it is plausible to think of *some* animals as members with rights to political status and membership as such. This plausibility opens their eligibility for protection via hate-speech laws under a Waldronian theory.

But even if it is accepted that some animals *should* be recognised as equal members of our communities, the fact remains that they are not. This would seem to mark an important difference in how we view the WDL and the HDL through Waldron’s theory. While the WDL’s leaflets act as an assault on dignity in our community here and now, the HDL’s do not. One response would argue that it does not matter whether animals are *recognised* as members of our society; they are members nonetheless.^62^ However, such a stark response is unnecessary. Waldron’s theory is not simply about vindicating what a society accepts, but helping realise what it ought to be.^63^ On his view, hate speech prohibits progress towards a well-ordered society, and laws prohibiting it can help to create the attitudes and environment necessary to uphold dignity for all.^64^ In response to those who argue against restrictions on hate speech, arguing that the views expressed should be left to wither away in the free marketplace of ideas, Waldron writes: ‘Societies do not become well-ordered by magic. The expressive and disciplinary work of law may be necessary as an ingredient in the change of heart within its racist citizens that a well-ordered society presupposes.’^65^ And if this logic works in the case of human members of our society, then it will work in the case of animal members. Their membership will not just happen by magic; the law—including prohibitions on speech acts like the HDL’s—can be a necessary ingredient in moving towards the good society.^66^

### D. The Harm of Undermining Assurance

There is a further Waldronian ground for differentiating between the WDL and the HDL, and it is one that resonates with our prior discussion of wounding. As we have seen, Waldron’s theory rests not only on members not having their dignity assaulted, but their being ‘assured’ of not having it undermined within their community. Waldron argues that it is vital that individuals can go about their daily lives secure in the knowledge that they will not have their standing undermined by speech that denigrates and abuses them. Most animals, however, cannot understand speech acts that attack their status, such as those of the HDL, making it unclear how they can be harmed by them.

Once again, however, we find that a closer reading of Waldron’s theory shows this objection to be misconceived. Waldron is quite explicit that the harms of hate speech cannot be reduced to the *feelings* they produce; rather, they are about the actual effects they have on individuals’ standing:

The distinction is in large part between objective or social aspects of a person’s standing in society, on the one hand, and subjective aspects of feeling, including hurt, shock, and anger, on the other. A person’s dignity or reputation has to do with how things are with respect to them in society, not with how things feel to them.^67^

None of this is meant to deny the felt aspects of assaults on dignity experienced by many humans, which are real and profound; instead, it is to say that it is the attack on dignity that constitutes the harm, not the feelings it produces. To illustrate his point, Waldron draws on the concepts of ‘degradation’ and ‘defamation’. In most cases, degrading treatment produces a great deal of hurt and shame. However, even in those exceptional cases where it does not, the harm is nonetheless real. And while defamation also usually results in feelings of distress, it is the assault on reputation that constitutes the harm, not the feelings the assault evokes.^68^

The harm that hate speech can have independently of any feelings of degradation on the part of the speech’s target can be illustrated by the opening examples in Waldron’s book. When he talks of the effect of hate speech, he talks not just of the effect it has on the target of the speech; there is a second audience. Racist graffiti, for example, speaks not simply to members of the racial minority, but to the racial majority, especially those who themselves harbour racist sentiments. To these people, Waldron claims, it says:

We know that some of you agree that these people are not wanted here. We know that some of you feel that they are dirty (or dangerous or criminal or terrorist). Know now that you are not alone. Whatever the government says, there are enough of us around to make sure these people are not welcome. There are enough of us around to draw attention to what these people are really like. Talk to your neighbors, talk to your customers. And above all, don’t let any more of them in.^69^

The standing of members of the minority is thus undercut, as—regardless of their own feelings on the matter—members of the majority are reminded and assured that members of the minority are not *really* members, that they do not *really* belong, that they are not *really* equals.

One problem with Waldron’s position here is that it might seem to be collapsing into a claim about ‘hostile environments’, whose problems as a justification of the discrepency we examined earlier. But even leaving that aside, there remain problems with using it to differentiate between the HDL’s and WDL’s leaflets. For if the harm of hateful speech lies in the *fact* that it undermines a group’s status, rather than the *feeling*s of the targeted group, then both leafleting campaigns must be censured. After all, the HDL do not speak to animals—they speak to humans, seeking to assure them of their superiority, of their right to use animals for their purposes, and so on. Since the HDL’s speech serves to provide arguments to undermine the worth and standing of animals, it straightforwardly seems to constitute an assault on dignity, and the type of harm that the law should be concerned with under these kinds of theories.

## 4. Conclusion

We have reviewed a range of reasons to think that those engaging in racist hate speech should be subject to legislative censure while those engaging in speciesist hate speech should not. We have found all such reasons wanting.

We accept that our arguments will not convince everyone. Readers who completely reject the idea of animal rights or animal membership, for example, will be able to find resources to justify treating the leaflets of the WDL and HDL very differently. But while there is not the room in this article to offer a full-blown defence of either animal rights or animal membership, we hope that they are sufficiently plausible for readers to accept that we have raised important questions about the scope of hate-speech laws.

Furthermore, we have not reviewed every possible theory outlining the wrongness of hate speech or the justifiability of hate-speech legislation. We have said nothing, for instance, about offence.^70^ However, we believe that we have addressed the most plausible ways that the discrepancy might be justified. Thus, though we allow that there may be some other way to justify the discrepancy between responses to racist and speciesist hate speech, we do not know what this would be.^71^

Finally, we acknowledge that even if the harmfulness of hate speech (racist *or* speciesist) provides a *prima facie* reason to support the criminalisation of said speech, it is plausible that, all things considered, criminalisation is not justified. Perhaps, for example, the harms of criminalisation would be significant enough to counterbalance the harms criminalisation seeks to avert. Or perhaps the harms of hate speech could be counterbalanced without the need for the drastic step of criminalisation, such as through counter-speech.^72^

With these caveats in mind, we conclude that we have found no good reason to endorse the discrepancy: *either* there is good reason to criminalise both racist and speciesist hate speech *or* neither should be criminalised.

## Footnotes

1 The WDL are fictional, but there have been many convictions in liberal states for the distribution of racist leaflets. Among the motley claims of convicted racists are implicit or explicit assertions of inequality between races, accusations of propagandising, and arguments about how one should prioritise the welfare of their favoured group. These are exactly the kinds of claims made by the WDL. For just one recent example, see *R v Sheppard & Whittle* (2010) EWCA Crim 65. For a more recent case which has resulted in much discussion, see ‘Britain First Leader and Deputy Leader Jailed for Hate Crimes’ (*BBC News*, 7 March 2018) <www.bbc.co.uk/news/uk-england-43320121> accessed 13 May 2020.

2 Public Order Act 1986, s 19.

3 Not all laws governing hate speech are criminal. For example, the law of tort could be leveraged against perpetrators of (putative or actual, criminal or non-criminal) hate speech. In the United States, where hate speech is not criminally sanctioned, some people targeted by hateful speech have successfully pursued damages under the tort of intentional infliction of emotional distress. See Alexander Brown, *Hate Speech Law* (Routledge 2015) 32–3, 52–3.

4 Joan Dunayer, ‘Sexist Words, Speciesist Roots’ in Carol J Adams and Josephine Donovan (eds), *Animals and Women: Feminist Theoretical Explorations* (Duke UP 1995).

5 The homophobic pastor may sincerely claim that he does not *hate* homosexuals, he simply wants to *save* them. And, as our opening examples show, hate speech can refer to written words just as readily as it can spoken words. See Jeremy Waldron, *The Harm in Hate Speech* (Harvard UP 2012) ch 3.

6 It is at least possible, for example, that we should protect animals from hate speech, but that (for whatever reason) none of these things constitute hate speech.

7 We are not committed to the claim that harm-based defences are necessarily tied up with consequentialist moral theory, but they *are* concerned broadly with the negative consequences of hate speech.

8 We thank both Jasmine Gunkel and an anonymous reviewer for this journal for pressing us on this issue.

9 John Stuart Mill, ‘On Liberty’ in John Gray (ed), *On Liberty and Other Essays* (first published 1859, OUP 2008) ch II.

10 cf Alexander Brown, ‘The Racial and Religious Hatred Act 2006: A Millian Response’ (2008) 11 Critical Review of International Social and Political Philosophy 1. Some have argued that Mill’s defence of free speech need not cover forms of speech—perhaps including instances of hate speech—that do not contribute to the development of deliberative capacities. See eg David O Brink, ‘Millian Principles, Freedom of Expression, and Hate Speech’ (2001) 7 LEG 119.

11 See John Rawls *Political Liberalism* (first published 1995, Columbia UP 2003) passim.

12 For a Rawlsian argument for restrictions on speech, see eg Matteo Bonotti, ‘Political Liberalism, Free Speech and Public Reason’ (2014) 14 European Journal of Political Theory 180. Bonotti ties his arguments about enforcing the Rawlsian duty of civility to Jeremy Waldron’s Rawlsian case for the criminalisation of hate speech, discussed later in the present article.

13 Federico Zuolo, *Animals, Political Liberalism and Public Reason* (Palgrave Macmillan 2020) 79–82.

14 Alasdair Cochrane, *Sentientist Politics: A Theory of Global Inter-Species Justice* (OUP 2018) 104.

15 Martha Nussbaum, *Frontiers of Justice: Disability, Nationality, Species Membership* (Harvard UP 2006) 388.

16 Cochrane (n 14) 105.

17 Zuolo (n 13) ch 4.

18 Chad Flanders, ‘Public Reason and Animal Rights’ in Marcel Wissenburg and David Schlosberg (eds), *Political Animals and Animal Politics* (Palgrave Macmillan 2014) 56.

19 Kimberly Smith, ‘A Public Philosophy for the Liberal Animal Welfare State’ in Robert Garner and Siobhan O’Sullivan (eds), *The Political Turn in Animal Ethics* (Rowman & Littlefield International 2016).

20 Alexander Brown, ‘The “Who?” Question in the Hate Speech Debate. Part 1. Consistency, Practical, and Formal Approaches’ (2016) 29 CJLJ 275, 301.

21 ibid 313.

22 ibid.

23 Iris Marion Young, *Justice and the Politics of Difference* (first published 1990, Princeton UP, 2011) 46.

24 Joanna Botha, ‘The Selection of Victim Groups in Hate-Crime Legislation’ (2019) 136 SALJ 781, 803.

25 Edward Ludwig and Dale Jamieson, ‘Animals as a Minority’ (1981) 5 Humanity & Society 246.

26 John Scott, ‘Minority Group’ in *A Dictionary of Sociology* (4th edn, OUP 2015).

27 Louis Wirth, ‘The Problem of Minority Groups’ in Ralph Linton (ed), *The Science of Man in the World Crisis* (OUP 1945).

28 Jon Garland, ‘It’s a Mosher Just Been Banged for No Reason: Assessing the Victimisation of Goths and the Boundaries of Hate Crime’ (2010) 17 International Review of Victimology 159.

29 Botha (n 24) 805.

30 Richard Delgado, ‘Words that Wound: A Tort Action for Racial Insults, Epithets, and Name-Calling’ (1982) 17 Harv CR-CL Law Rev 133.

31 We allow that some speech acts targeting some animals could be wounding. Animals could potentially be wounded by the *action* of speech rather than the *content* of speech. For example, an animal may be wounded by sudden loud shouting, regardless of what is said—a surprise yell of ‘Precious puss!’ may well be much more disturbing than a muttered ‘Repulsive creature!’ Perhaps, too, some animals could come to associate particular words with unpleasant experiences—a dog brutally ‘disciplined’ could perhaps be wounded by the phrase ‘Bad dog!’ But this is all far removed from the kinds of cases we are presently exploring.

32 Joel Feinberg, *Moral Limits of the Criminal Law.* *Volume 2.* *Offense to Others* (OUP 1988) 30.

33 Adapted from ibid, emphasis Feinberg’s.

34 Adapted from ibid 30–1, emphasis added.

35 Leaving aside the possibility that the individuals are wounded by the way the message is delivered as a different matter. See n 31.

36 Perhaps *others* will be wounded by awful things said about these people—but this could be true of animals, too. In any case, it seems to miss the point. One of the things that favours the wounding-words account is the intuitive appeal of the thought that hate speech is wrong *because of wrongs done to those it targets*.

37 Consider also fair assessments (say, frank criticism of someone’s too-ambitious career plans), challenges of deeply held beliefs (such as ‘blasphemous’, though dispassionate and objective, rejections of the sanctity of certain figures), and innocuous trigger words in even placid environments (such as words that remind individuals of traumatic experiences). If the hurt felt in response to hate speech is enough to justify sanction of that sort of speech, why not the hurt felt in response to these other kinds of speech? Again, we are not claiming that this issue cannot be overcome, simply observing that the account’s subjectivity raises some interesting questions.

38 Jeffrey Howard, ‘Free Speech and Hate Speech’ (2019) 22 Annual Review of Political Science 93, 103.

39 See Brown, ‘The Racial and Religious Hatred Act 2006’ (n 10).

40 Rae Langton, ‘The Authority of Hate Speech’ in John Gardner, Leslie Green, and Brian Leiter (eds), *Oxford Studies in Philosophy of Law*, vol 3 (OUP 2018).

41 Brown, ‘The Racial and Religious Hatred Act 2006’ (n 10) 13.

42 Robert Mark Simpson, ‘Dignity, Harm and Hate Speech’ (2013) 32 Law and Philosophy 701, 723.

43 This number refers to the number of terrestrial vertebrates killed for food according to the Food and Agricultural Organisation of the UN <http://www.fao.org/faostat/>. It does not include the many more non-terrestrial animals and invertebrates killed for food. Nor does it include animals killed for other purposes.

44 Dinesh Joseph Wadiwel, ‘The War Against Animals: Domination, Law and Sovereignty’ (2009) 18 GLR 283.

45 Notwithstanding the point that we remain agnostic on the question of whether there should be laws against hate speech at all.

46 Waldron’s use of *dignity* is rather unusual, and very much relates to political status grounded in membership of a community as opposed to moral status grounded in the type of being that one is.

47 Waldron, *The Harm in Hate Speech* (n 5) 57.

48 ibid 61.

49 ibid 85.

50 ibid 5–6.

51 ibid 101–3.

52 Gary Francione, *Animals, Property and the Law* (Temple UP 1995).

53 See eg ‘Animals in France Finally Recognized as Living, Sentient Beings’ (*RT News*, 29 January 2015) <https://on.rt.com/fv7zwj> accessed 6 October 2020.

54 See art 13 of the Treaty on the Functioning of the European Union <eur-lex.europa.eu/resource.html> accessed 6 October 2020.

55 See Jessica Eisen and Kirsten Stilt (2016) ‘Protection and Status of Animals’ in Rüdiger Wolfrum, Frauke Lachenmann, and Rainer Grote (eds), *Max Planck Encyclopedia of Comparative Constitutional Law* <oxcon.ouplaw.com/home/MPECCOL> accessed 6 October 2020.

56 This is based on Waldron’s own writings on animals. See eg Jeremy Waldron, *One Another’s Equals: The Basis of Human Equality* (Harvard UP 2017).

57 Sue Donaldson and Will Kymlicka, *Zoopolis: A Political Theory of Animal Rights* (OUP 2011).

58 Claudio Lopez-Guerra, ‘Enfranchising Minors and the Mentally Impaired’ (2012) 38 Social Theory & Practice 115.

59 Cochrane (n 14).

60 Elizabeth Anderson, ‘Animal Rights and the Values of Nonhuman Life’ in Cass Sunstein and Martha Nussbaum (eds), *Animal Rights: Current Debates and New Directions* (OUP 2005); Kimberly Smith, *Governing Animals: Animal Welfare and the Liberal State* (OUP 2012); Laura Valentini, ‘Canine Justice: An Associative Account’ (2013) 62 Political Studies 37.

61 Will Kymlicka, ‘Social Membership: Animal Law Beyond the Property/Personhood Impasse’ (2017) 40 Dalhousie Law Journal 123.

62 This is explicitly argued by Donaldson and Kymlicka in ‘Animals in Political Theory’ in Linda Kalof (ed), *The Oxford Handbook of Animal Studies* (OUP 2017); see also Smith, *Governing Animals* (n 60).

63 Waldron, *The Harm in Hate Speech* (n 5) 79–82.

64 cf Simpson (n 42) 722–3.

65 Waldron, *The Harm in Hate Speech* (n 5) 81.

66 Their being necessary need not mean that it is wise to introduce them immediately. Again, it may be helpful to view our arguments as philosophy of law for a more ideal world. Perhaps, for example, we find the liberation of members of racial minorities and of nonhuman animals on different timelines (a phrase suggested to us by Sue Donaldson). Perhaps we are at the point where the institution of hate speech laws can help push us towards just race relations, but we are not at the same point when it comes to interspecies relations. For instance, we may think that there is a relevant difference in practice (if not in theory) between the levels of *responsibility* that can be attributed to individuals for engaging in hate speech targeting members of racial minorities and hate speech targeting animals. We may think it questionable to criminally sanction people for expressing views that are very commonly held in their society, *even if* said expression is just as harmful as the expression of certain views *not* commonly held that *are* criminally sanctioned. We thank Will Kymlicka for prompting these ideas.

67 Waldron, *The Harm in Hate Speech* (n 5) 106.

68 ibid 109–10.

69 ibid 2–3.

70 Though the HDL’s conduct, unlike the WDL’s conduct, is unlikely to cause a great deal of offence, the offensiveness of hate speech is rarely seriously proposed as a reason to criminalise it. Joel Feinberg, who argues for the legitimacy of the criminalisation of offensive conduct, would likely not support criminalising the conduct of the WDL anyway, as he sees the sincere and civil expression of opinion as ‘a kind of trump card’. No matter how odious an opinion, he argues, expressing it is permissible. See Feinberg (n 32) 39.

71 It is worth acknowledging, too, that this methodology may be the wrong way to go about this. Puzzling through the many and various ways that hate-speech law could be justified (and the many and various apparent counter-examples to justifications for hate-speech law) might miss the significance of hate speech. Perhaps, it could be countered, it is the *whole* of (racist, sexist) hate speech that justifies censure, rather than any particular constituent part. We are reminded of the words of Robert Nozick, a noted philosopher sympathetic to the plight of animals who nonetheless questioned the anti-speciesism of animal rights theory. ‘We think of humans,’ Nozick wrote, ‘as part of the multifarious texture of human history and civilizations, human achievements, and human family relations. Animals … we see against a different background and texture. The differences are enormous and endless.’ The mistake, Nozick argues, that animal-rights theorists make is assuming that ‘something much simpler than the total differences between two rich tapestries (one richer than the other) will, by itself, constitute the morally relevant difference’. It could be that something similar is true of hate speech: there *is* a difference between anti-animal hate speech and (*inter alia*) racist hate speech, but it cannot be stated succinctly. This challenge, Nozick fears, is unanswerable: philosophers simply have not yet developed the tools to assess *wholes* in this way. See Robert Nozick, *Socratic Puzzles* (Harvard UP 1997) 308–9. For more on Nozick and animals, see Josh Milburn, ‘Robert Nozick on Nonhuman Animals: Rights, Value and the Meaning of Life’ in Andrew Woodhall and Gabriel Garmendia da Trindade (eds), *Ethical and Political Approaches to Nonhuman Animal Issues* (Palgrave Macmillan 2017).

72 cf Corey Brettschneider, *When the State Speaks, What Should It Say?* (Princeton UP 2012).

